# Thoracic radiotherapy plus Durvalumab in elderly and/or frail NSCLC stage III patients unfit for chemotherapy - employing optimized (hypofractionated) radiotherapy to foster durvalumab efficacy: study protocol of the TRADE-hypo trial

**DOI:** 10.1186/s12885-020-07264-8

**Published:** 2020-08-26

**Authors:** Farastuk Bozorgmehr, Inn Chung, Petros Christopoulos, Johannes Krisam, Marc A. Schneider, Lena Brückner, Daniel Wilhelm Mueller, Michael Thomas, Stefan Rieken

**Affiliations:** 1 Department of Thoracic Oncology, Thoraxklinik at University Hospital of Heidelberg, Röntgenstraße 1, 69126 Heidelberg, Germany; 2 Translational Lung Research Center Heidelberg TLRCH, Member of the German Center for Lung Research DZL, Im Neuenheimer Feld 156, 69120 Heidelberg, Germany; 3 Institute of Medical Biometry and Informatics, University Hospital of Heidelberg, Im Neuenheimer Feld 130.3, 69120 Heidelberg, Germany; 4 Thoraxklinik at University Hospital of Heidelberg, Translational Research Unit (STF), Röntgenstraße 1, 69126 Heidelberg, Germany; 5 Institute of Clinical Cancer Research IKF GmbH at Northwest Hospital, Steinbacher Hohl 2-26, 60488 Frankfurt am Main, Germany; 6 Department of Radiation Oncology, University Medical Center Göttingen, Robert-Koch-Str. 40, 37075 Göttingen, Germany

**Keywords:** Non-small cell lung cancer, NSCLC, Radioimmunotherapy, Immune checkpoint inhibition, Anti-PD-L1 monoclonal antibody, Hypofractionated radiation, Geriatric risk profile

## Abstract

**Background:**

Non-small cell lung cancer is the most common cause of cancer death worldwide, highlighting the need for novel therapeutic concepts. In particular, there is still a lack of treatment strategies for the group of elderly and frail patients, who are frequently not capable of receiving standard therapy regimens. Despite comprising the majority of lung cancer patients, this group is underrepresented in clinical trials. This applies also to elderly and frail patients suffering from unresectable stage III NSCLC, who are unfit for chemotherapy, and, therefore, cannot receive the standard therapy comprising of radiochemotherapy and the recently approved subsequent durvalumab consolidation therapy. These patients often receive radiotherapy only, which raises the concern of undertreatment. The TRADE-hypo trial aims at optimizing treatment of this patient group by combining radiotherapy with concomitant durvalumab administration, thereby employing the immune-promoting effects of radiotherapy, and determining safety, feasibility, and efficacy of this treatment.

**Methods/ design:**

In this prospective phase II clinical trial, durvalumab therapy will be combined with either conventionally fractionated (CON-group) or hypofractionated (HYPO-group) thoracic radiotherapy. A safety stop-and-go lead-in phase will assess safety of hypofractionated radiotherapy with respect to severe pneumonitis in small patient cohorts before opening full enrollment. Tumor tissue, blood and stool samples will be collected before and during the study period to investigate the immunological mechanisms responsible for checkpoint inhibitor efficacy and immune-promoting effects of radiotherapy.

**Discussion:**

Preclinical data suggests that irradiation-induced immunogenicity can be further increased if applied in a hypofractionated setting, potentially boosting the expected synergistic effect with immune checkpoint inhibition in restoring the immune anti-tumor response. If proven safe and efficient, a hypofractionated radiation schedule can provide a considerably more practicable option for the patient. Taking into consideration the intend to develop a combination treatment strategy that can be made available to patients soon after proving to be efficient and the potentially elevated toxicity of a hypofractionated radiotherapy approach, this trial was designed as a two-trials-in-one design. An accompanying translational research program is planned striving to gain insights into the tumor-host biology and to identify suitable biomarkers to predict therapy response.

**Trial registration:**

Clinicaltrials.gov, NCT04351256. Registered 17 April 2020,

Eudra-CT, 2019–002192-33. Registered 24 October 2019,

## Background

Lung cancer is the most common cause of cancer death worldwide, with non-small cell lung cancer (NSCLC) representing 80–90% of cases [1, 2]. Improving therapeutic strategies is thus of imminent importance, especially considering elderly and frail patients. With a median age of about 70 years at diagnosis lung cancer clearly is a disease of the elderly, yet this group is underrepresented in clinical trials, and these patients are frequently not capable of receiving standard treatment protocols due to age- and tobacco-associated comorbidities [3–5].

In recent years, the advent of immunotherapy has paved the way for novel therapeutic concepts, including the combination of radiotherapy with immune checkpoint inhibition (e.g. Programmed cell death/ -ligand 1; PD-1/ PD-L1). This approach is of particular interest as it utilizes synergistic effects: While immune checkpoint inhibitors can restore the patients’ antitumor immunity through T cell activation, radiotherapy may further boost immune-mediated anti-cancer mechanisms by exposing tumor-associated antigens and by attracting both immunocompetent antigen-presenting cells and tumoricidal effector cells [6, 7]. Indeed, for patients with unresectable stage III NSCLC, the PACIFIC trial has revealed a profound clinical benefit treatment with the anti-PD-L1 monoclonal antibody durvalumab after chemoradiotherapy with remarkably low toxicities [8, 9]. As a result, sequential treatment with durvalumab after chemoradiotherapy has become the new standard treatment for locally advanced, unresectable NSCLC. However, about 20% of patients do not receive chemotherapeutic agents, presumably due to significantly higher rates of age- and comorbidity-related adverse events (AE) under chemoradiotherapy [5, 10]. Thus, elderly and frail patients often receive radiotherapy alone, raising the serious concern of undertreatment and the need for new therapeutic concepts [4, 5].

Considering the immune-promoting effects of radiotherapy, a combination with durvalumab therapy may improve response rates in these potentially undertreated patients. Moreover, if applied early, concomitant local radiotherapy with systemic immunotherapy may particularly increase control of distant micrometastases. Preclinical data suggest that irradiation-induced immunogenicity can even be further increased if applied in a hypofractionated setting with single doses ≥3 Gray (Gy), in line with a radiation dose-dependent abscopal effect [11–14]. While a hypofractionated radiation schedule is also considerably shorter and more convenient for the patient, safety of concurrent immunoradiotherapy is a concern, as both therapy modalities may cause severe pneumonitis.

In this prospective phase II clinical trial, we therefore aim to determine feasibility and treatment efficacy of durvalumab treatment combined with thoracic radiotherapy (TRT) in previously untreated NSCLC stage III patients unable to receive radiochemotherapy. Striving to develop a combination treatment strategy that, if proven safe and efficient, can be quickly made available to patients, a two-trials-in-one design was chosen that combines durvalumab with either conventionally fractionated (CON-group) or hypofractionated thoracic radiotherapy (HYPO-group). This study not only aims to increase the efficacy of radiotherapy by utilizing the immune-sensitizing effects elicited by PD-L1 inhibition, but will also provide biomaterials that will be analyzed with respect to immunological mechanisms responsible for checkpoint inhibitor efficacy and immune-promoting effects of radiotherapy as well as potential biomarkers.

## Methods/design

### Study design

**Fig. 1:**
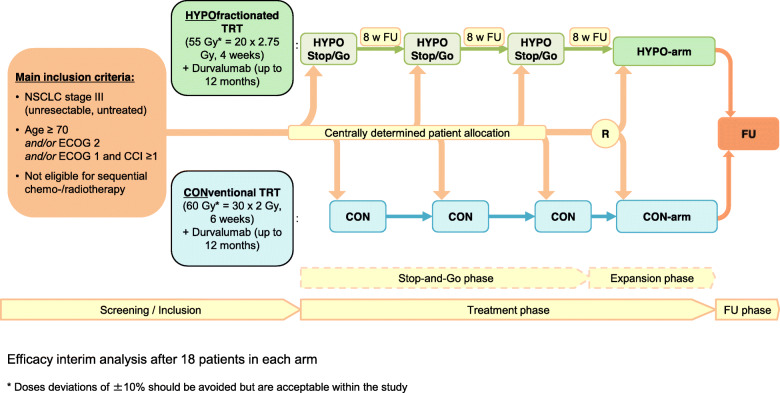
Study design of the TRADE-hypo trial. Patients will be enrolled according to eligibility criteria and treated with either a hypofractionated TRT regimen (HYPO-group) or conventionally fractionated TRT (CON-group) in combination with durvalumab. For the HYPO-group, a safety stop-and-go phase with a 6 + 6 design precedes full enrollment. Whenever this arm is open for recruitment, patients will be allocated to this arm until the cohort is closed; whenever HYPO-arm is closed for Stop/ Go decision evaluation based on the toxicity assessment of this regimen 8 weeks after the end of TRT, patients are allocated to the CON-arm. When the study proceeds to expansion phase, patients will be allocated to treatment arms by randomization using “biased coin” algorithm. An efficacy interim analysis will be performed after 18 patients have been enrolled in each arm

**Fig. 2:**
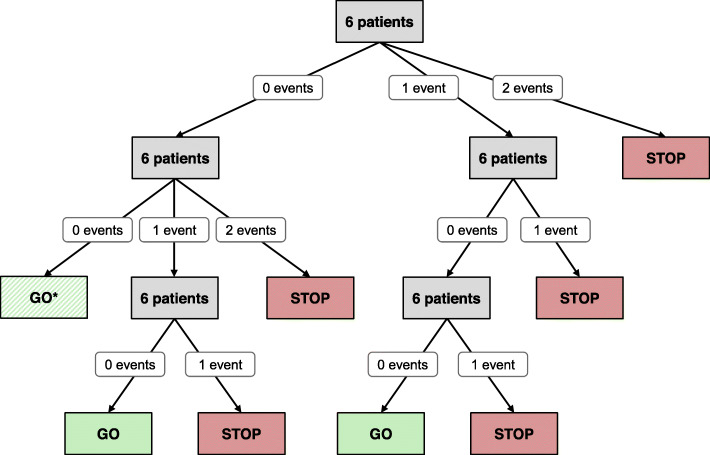
Cohort design of the safety stop-and-go lead-in phase (HYPO-group). The safety lead-in phase follows a 6 + 6 design in order to carefully evaluate the toxicity of the treatment in the HYPO-group with respect of the occurrence of a grade 3/4 pneumonitis (“event”) within 8 weeks after the end of TRT. Two events in the first six patients, two events in the first 12, or two events in the first 18 patients will result in termination of the HYPO-group (“Stop”). If no event is observed within the first two safety cohorts, i.e. the first 12 patients, the HYPO-arm will be opened for full enrollment with close toxicity assessment with respect to pneumonitis grade 3/4, and terminated as soon as two events are reported within the subsequent six patients (“Go*”). Full enrollment in the HYPO-arm will only take place if the criteria for the non-toxicity scenario are met, i.e. ≤ 1 event in *n* = 18 patients (“Go”)

The TRADE-hypo trial is a prospective, randomized, open-label, multicenter, phase II trial with a safety stop-and-go lead-in phase (Fig. 1). During the lead-in phase, patients in the HYPO-group, who will receive durvalumab in combination with hypofractionated thoracic radiotherapy, will be closely evaluated with regard to toxicity (defined as pneumonitis ≥ grade 3 within 8 weeks after radiotherapy) in small cohorts (*n* = 6) before proceeding with full enrollment into this arm (Fig. 2).

### Study setting

The TRADE-hypo trial will recruit patients from 17 participating centers across Germany over a period of 20 months. Start of recruitment was planned for April 2020, but was delayed to May 2020 due to the Covid-19 pandemic. A full list of sites can be obtained at clinicaltrials.gov (NCT04351256).

### Study objectives

The primary objective of this study is to evaluate safety and tolerability of conventionally fractionated (CON-group) and hypofractionated (HYPO-group) TRT in combination with durvalumab in patients with unresectable stage III NSCLC unfit for chemotherapy. Moreover, efficacies of the two modes of radiotherapy will be evaluated with respect to response rates. Further parameters will be determined in order to assess efficacy, safety, and quality of life (QoL) in both treatment arms by recording incidence and severity of adverse events (AEs) as well as specific laboratory abnormalities.

Exploratory endpoints include assessment of vulnerability and analyses of tumor tissue, blood, and stool samples that are collected during the clinical trial with respect to treatment-induced changes and immune-related biomarkers.

### Characteristics of participants

A total of 88 patients will be included into this study. Patients potentially eligible for trial inclusion will be approached and asked to participate as they present in the clinic. Before a patient’s participation in the clinical study, the investigator must obtain written informed consent.

Each participant must be eligible regarding all inclusion and exclusion criteria set for this trial (Table 1). Key inclusion criteria include diagnosis of unresectable stage III NSCLC and non-feasibility of sequential chemoradiotherapy due to increased (oxygen-independent) vulnerability as reflected by fulfilling at least one of the following criteria: (i) Performance status 2 (Eastern Cooperative Oncology Group [ECOG] scale), (ii) ECOG 1 and Charlson comorbidity index (CCI) ≥ 1, or (iii) age ≥ 70. Moreover, participants must have at least one measurable site of disease as defined by RECIST 1.1, as well as adequate bone marrow, hepatic and renal function. Patients having received prior immunotherapy, other investigational agents or thoracic radiotherapy within the past 5 years will be excluded from the study. Additionally, participants must not have an active or recent history of a known or suspected autoimmune disease or any other medical condition conflicting with the study interventions, and not have used immunosuppressive medication. For a full list of the inclusion and exclusion criteria see Table 1.

**Table 1:** Complete list of inclusion and exclusion criteria

**Inclusion criteria**	
• Fully-informed written consent and locally required authorization obtained from the patient/ legal representative prior to performing any protocol-related procedures, including screening evaluations. • Age ≥ 18 years. • Histologically documented diagnosis of unresectable stage III NSCLC. • Non-feasibility of sequential chemo−/radiotherapy as determined by the site’s multi-disciplinary tumor board; if there is no tumor board, then this decision will be made by the investigator in consultation with a radiation oncologist, if the investigator is not a radiation oncologist; or by the investigator in consultation with an oncologist, if the investigator is not an oncologist. • Fulfills at least one of the following criteria: ○ ECOG 2 ○ ECOG 1 and CCI ≥ 1 ○ Age ≥ 70 years • Must have a life expectancy of at least 12 weeks. • FEV1 ≥ 40% of predicted • DLCO ≥40% of predicted • FVC or VC ≥ 70% of predicted • At least one measurable site of disease as defined by RECIST 1.1 criteria. • Adequate bone marrow, renal, and hepatic function • Female patients with reproductive potential must have a negative urine or serum pregnancy test within 7 days prior to start of trial. • Evidence of post-menopausal status or negative urinary or serum pregnancy test for female pre-menopausal patients. • The patient is willing and able to comply with the protocol for the duration of the study, including hospital visits for treatment and scheduled follow-up visits and examinations.	
**Exclusion criteria**	
• Concurrent enrollment in another clinical study or enrollment within 21 days prior to first dose of treatment, unless it is an observational (non-interventional) clinical study, or during the follow-up period of an interventional study. • Prior immunotherapy or use of other investigational agents. • History or current radiology suggestive of interstitial lung disease. • Oxygen-dependent medical condition. • Any concurrent chemotherapy, investigational product (IP), biologic, or hormonal therapy for cancer treatment. • Prior thoracic radiotherapy within the past 5 years before the first dose of study drug. • Major surgery within 4 weeks prior to enrollment into the study; patients must have recovered from effects of any major surgery. Local non-major surgery for palliative intent is acceptable. • Active or prior documented autoimmune or inflammatory disorders, with the following exceptions: Patients with vitiligo or alopecia, patients with hypothyroidism stable on hormone replacement, or any chronic skin condition that does not require systemic therapy. Patients without active disease in the last 5 years may be included but only after consultation with the study physician. • Active, uncontrolled inflammatory bowel disease. Patients in stable remission for more than 1 year may be included. • Uncontrolled intercurrent illness, including but not limited to, ongoing or active infection, symptomatic congestive heart failure, uncontrolled hypertension, unstable angina pectoris, uncontrolled cardiac arrhythmia, interstitial lung disease, gastrointestinal conditions associated with diarrhea, or psychiatric illness/social situations that would limit compliance with study requirement, substantially increase risk of incurring AEs or compromise the ability of the patient to give written informed consent. • History of another primary malignancy except for a malignancy that has been treated with curative intent and was not active for ≥5 years before the first dose of IP and of low potential risk for recurrence or adequately treated non-melanoma skin cancer or lentigo maligna without evidence of disease or adequately treated carcinoma in situ without evidence of disease • History of leptomeningeal carcinomatosis • History of active primary immunodeficiency • History of allogenic organ or tissue transplantation. • Clinical diagnosis of active tuberculosis. • Positive testing for hepatitis B virus surface antigen or hepatitis C virus RNA indicating acute or chronic infection or for human immunodeficiency virus. • Current or prior use of immunosuppressive medication within 14 days before the first dose of durvalumab. The following are exceptions to this criterion: Intranasal, inhaled, topical steroids, or local steroid injections; systemic corticosteroids at physiologic doses not to exceed 10 mg/day of prednisone or its equivalent; steroids as premedication for hypersensitivity reactions • Receipt of live attenuated vaccine within 30 days prior to the first dose of IP. • Female patients who are pregnant or breastfeeding or male or female patients of reproductive potential who are not willing to employ effective birth control. • Known allergy or hypersensitivity to any of the IPs or any of the constituents of the product. • Any co-existing medical condition that in the investigator’s judgement will substantially increase the risk associated with the patient’s participation in the study. • Patient who has been incarcerated or involuntarily institutionalized by court order or by the authorities § 40 Abs. 1 S. 3 Nr. 4 AMG. • Patients who are unable to consent because they do not understand the nature, significance and implications of the clinical trial and therefore cannot form a rational intention in the light of the facts [§ 40 Abs. 1 S. 3 Nr. 3a AMG].	

### Treatment

#### Durvalumab

Patients will be enrolled based on the in−/ exclusion criteria. Two treatment groups will be evaluated: One group will receive durvalumab immunotherapy combined with conventionally fractionated TRT (CON-group) and the other one with hypofractionated TRT (HYPO-group). In both groups, durvalumab will be administered intravenously at a fixed dose of 1500 mg on day 1 and every 4 weeks thereafter for a maximum of 12 months (maximum 13 doses, last dose at week 49) until confirmed disease progression, inacceptable toxicity, withdrawal of consent or end of the study (Fig. 1 and Table 2).

**Table 2:** Schedule of assessments

Procedure / Point in time	Screening	Treatment Cycles (Q4W)						EOT	Post-Treatment	
	Screening	C1D1	C1D4	C2D1	C3D1	C4D1	C5D1-C13D1	EOT	Safety FU	FU (Q12W)
Informed Consent, eligibility criteria, demographics, medical and history	X									
Allocation/ Randomization	X									
Prior and Concomitant Medication Review	X	X		X	X	X	X			
Durvalumab administration		X		X	X	X	X			
Radiotherapy (CON^a^ or HYPO^b^)			X							
AEs	X	X		X	X	X	X	X	X	X
Full Physical Examination	X							X		
Directed Physical Examination		X		X	X	X	X		X	
Vital Signs, O_2_ Saturation, and Weight	X	X		X	X	X	X	X	X	
Pulmonary function tests	X			(X)^c^	(X) ^c^	together with staging		X	X	
12-lead ECG	X	Whenever clinically indicated								
ECOG Performance Status	X	X		X	X	X		X		
Pregnancy Test, CBC with Differential, Serum Chemistry Panel, Thyroid function test	X	X		X	X	X	X	X		
HBV/ HCV	X									
Urinanalysis	X	Whenever clinically indicated								
Tumor Imaging	X			(X)^d^	(X)^d^	X^e^	Q8W^e^	(X)^f^		X^g^
FACT-L and G8 screening questionnaire	X	X		X	X	together with staging		X	X	(X)^h^
Tissue	X	Optional: Re-Biopsy at time of progression								
Blood and stool	X^i^	X^i^		X		X		X		

^a^CON-group radiation scheme: Patients receive conventional fractions of 30 × 2 Gy (60 Gy) within 6 weeks of TRT to be started within 72 h after start of durvalumab treatment

^b^HYPO-group radiation scheme: Patients receive hypofractionated thoracic radiotherapy consisting of 20 × 2,75 Gy (55 Gy) within 4 weeks of TRT to be started within 72 h after start of durvalumab treatment

^c^To be performed on C2D1 and C3D1 if in accordance with local standard

^d^Chest X-ray to be performed on cycles 2 and 3 if in accordance with local standard

^e^First on-study CT imaging to be performed 12 weeks after first durvalumab administration. Further on-study imaging to be performed Q8W (56 days ±7 days)

^f^Only applicable if EOT not according to already detected disease progression

^g^In patients with EOT not due to disease progression tumor imaging will be performed until the start of a new anticancer treatment, disease progression, death, withdrawal of consent, or the end of the study

^h^Questionnaires will be collected until disease progression only and may be collected by telephone calls

^i^Biomarker sample to be taken prior to first study drug medication either during screening or C1D1 visit

#### Radiotherapy

All patients are subjected to preparation of individual positioning devices and CT-based planning. Motion management may comprise either 4D-CT or midbreathing CT image acquisition. Further imaging modalities, such as FDG-PET/CT, may be used when deemed necessary. Gross tumor volumes (GTV) will be contoured and expanded by adequate clinical (CTV) and planning (PTV) safety margins in order to account for subclinical disease and positioning errors. No elective nodal irradiation will be performed. As for organs at risk, both lungs, spinal cord, heart and esophagus must be contoured. In the HYPO-arm, no more than 30% of “both lungs minus GTV” should receive > 20 Gy; in the CON-arm, no more than 35% of “both lungs minus GTV” should receive > 20 Gy.

In the HYPO-arm, 20 fractions of 2.75 Gy will be administered (total dose 55 Gy, corresponding to 70 GyBED, α/β = 10). In the CON-arm, 30 fractions of 2 Gy will be administered (total dose 60 Gy, corresponding to 72 GyBED, α/β = 10). Dose deviations of ±10% are acceptable, when clinically indicated. Radiotherapy is scheduled to start within 72 h after the first administration of durvalumab. Dose prescription will follow international reports (ICRU 50, 62 and 83). Both 3D-conformal and modulated photon radiation techniques, such as IMRT and VMAT/RapdArc, are acceptable. All participating institution are encouraged to perform regular, if no daily, positioning control using either portal imaging or on-board-CT devices.

### Study procedures

In order to minimize the risk exposure of patients when subjected to the hypofractionated radiation regimen, a safety lead-in phase with stop-and-go design will precede full enrollment into the HYPO-group (Fig. 2). Toxicity will be evaluated with a 6 + 6 design that is based on the statistical assumption that ≤1 event in *n* = 18 patients conforms to a non-toxicity scenario, with “event” being defined as the occurrence of pneumonitis grade ≥ 3 within 8 weeks after end of TRT. Consequently, two events in the first six patients or two events in the first 12 or two events in the first 18 patients will result in termination of the HYPO-group (Fig. 2).

During this safety stop-and-go phase, patients will be allocated to treatment arms as follows: While the HYPO-arm is recruiting, patients will exclusively enter this treatment group. During safety evaluation of the six patients of a HYPO-cohort (Stop/ Go decision), patients will be allocated to the CON-group only. If safety criteria in the HYPO-cohort are met, the HYPO-arm will be reopened to continue toxicity assessment, and patients will solely be allocated to this arm (Fig. 1). In order to avoid any bias, patients will be allocated centrally by the study management during this phase. If the non-toxicity criteria in the safety cohorts are met, the study will proceed to the expansion phase, and remaining patients will be randomized into the two treatment arms using an interactive web response system integrated in the electronic case report form (eCRF). A possibly uneven distribution of patients between the treatment groups at this stage will be compensated by a randomization strategy based on the “biased coin” method [15, 16]. In the randomization phase, patients will be stratified according to tumor stage (stage IIIA vs. stage IIIB/IIIC).

In total, 44 patients will be enrolled per group. After *n* = 18 patients have been enrolled to the HYPO- or CON-treatment arm, respectively, an interim efficacy analysis for the respective arm will be conducted based on the objective response rate (ORR) at 12 weeks after first durvalumab administration. In case of insufficient efficacy of one of the arms (i.e., the number of patients who have achieved a response is eight out of 18 or lower) this treatment arm will be terminated. Recruitment will be halted until results of the interim efficacy analysis are available.

Tumor response will be assessed according to RECIST 1.1 by CT and/ or MRI scans at baseline, 12 weeks after durvalumab initiation and then every 8 weeks. Safety measures will include physical examinations, performance status (ECOG), clinical laboratory profiles and continuous assessments of AEs. An overview of all study procedures is presented in Table 2.

An Independent Safety Monitoring Board (ISMB) will ensure the continued safety of participants throughout the trial. Data management and data quality assurance will be conducted following the Standard Operational Procedures of the Institute of Clinical Cancer Research IKF at Northwest Hospital GmbH (Frankfurt, Germany). An eCRF will be carefully maintained for each participant for data collection, also reporting serious and non-serious AEs following the common criteria for adverse events (CTCAE) version 5.0 throughout the entire trial. After the end of the study, participants will be proactively followed up regarding treatment-related AEs until resolved, returned to baseline or considered irreversible, until lost to follow-up, or withdrawal of study consent. All subjects will be followed for survival. Patients who decline to return to the site for evaluations will be offered a follow-up (FU) by phone every 3 months as an alternative. At the end of the study treatment, the investigators are responsible for the further treatment of the patient and must ensure that he or she receives appropriate standard of care or other appropriate therapies.

### Sampling for translational research

If patients participate in the translational research program, blood samples will be collected prior to cycles 1, 2 and 4 and at the time of disease progression (or end of treatment, EOT) along with stool samples (Table 2). Samples of untreated tumor lesions serving as baseline specimens will be collected as paraffin-embedded tissue. If re-biopsies are taken at the time of progression, samples should also be also submitted for translational research.

### Study endpoints

The primary endpoint of the study will be toxicity, defined by the occurrence of treatment-related pneumonitis grade ≥ 3. The ORR evaluated 12 weeks after first durvalumab administration (according to RECIST 1.1) is set as the primary efficacy endpoint. Secondary endpoints of the study comprise the occurrence of treatment-related AEs and serious AEs (SAEs), frequency of prespecified abnormal laboratory parameters, progression-free survival (PFS) and duration of clinical benefit, metastasis-free survival, overall survival (OS), and QoL.

Patient vulnerability and its association with survival and outcome will be assessed as an exploratory endpoint. To this end, the G8-screening questionnaire, a simple and fast screening tool for identifying geriatric risk profiles with a strong prognostic value for functional decline and OS in older populations with cancer, will be used [17]. Furthermore, biomaterials will be collected during the trial for correlation analyses on selected molecular parameters and clinical data in order to identify molecular biomarkers predictive for response to therapy. This approach is deemed appropriate to obtain hypothesis-generating data for future research due to the explorative character of this trial.

### Statistical analysis

#### Sample size justification

##### Safety run-in phase (HYPO-group)

With regard to the pneumonitis grade ≥ 3 rate, this phase is designed to distinguish between a toxicity scenario (pTox = 0.15) and a non-toxicity scenario (pTox = 0.035). A sample size of *n* = 18 will yield a probability of 0.78 to correctly detect the toxicity scenario, while the non-toxicity scenario will correctly be detected with a probability of 0.87. These probabilities are based on the decision rule that, if the number of patients with pneumonitis grade ≥ 3 in this cohort is ≥2, recruitment to the HYPO-group will be terminated.

##### Interim efficacy analysis regarding ORR and expansion phase

An interim efficacy analysis based on the ORR will be conducted after *n* = 18 patients in each arm have completed radiotherapy and the 18th patient has undergone first radiographic assessment (at 12 weeks after first durvalumab dose).

Previously, an ORR of 45% after radiotherapy alone has been reported in a Japanese population of elderly patients with unresectable stage III NSCLC [18]. Based on this and the observation that Asian ethnicity is associated with a favorable prognosis, we assume for the TRADE-hypo trial that an ORR higher than 0.42 in both treatment arms can be demonstrated, i.e. the null hypotheses for arm HYPO and CON are defined as H_0_^HYPO^: π ^HYPO^ ≤ 0.42 and H_0_^CON^: π ^CON^ ≤ 0.42, where π ^HYPO^ and π ^CON^ denote the actual ORR in arm HYPO and CON, respectively [19, 20]. Under the alternative hypothesis, it is assumed that both π ^HYPO^ and π ^CON^ amount to 0.60. Using an optimal Simon’s two-stage design with a one-sided significance level of α = 0.10 and a power of 1-β = 0.80 for each hypothesis test, *n* = 40 patients per arm are required, while the interim analysis is conducted after n = 18 patients per arm have been recruited to the trial. After successfully passing the safety analysis in the HYPO-group, if among 18 patients in the HYPO- or CON-arm, the number of patients who have achieved a response is eight or lower, the respective arm will be closed. Otherwise, an additional number of 22 patients will be enrolled into the respective arm. The null hypothesis of the respective arm can be rejected if at least 21 of all 40 patients per arm achieve a response. Sample size calculation was done using the R package “OneArmPhaseTwoStudy” [21].

To account for an estimated dropout rate of 10%, four patients will additionally be recruited to each treatment arm. Deviations from planned sample sizes will be handled as described by Englert & Kieser, allowing strict control of the aspired type I error rate in each arm [22].

##### Methods of statistical analysis

The primary population for evaluating all efficacy endpoints and subject characteristics is defined as all patients enrolled according to initial allocation/randomization (intention-to-treat population, IIT). Secondary efficacy analyses will be carried out on the per-protocol (PP) population. The safety population comprising all patients who received at least one dose of the study medication will be used for all safety endpoints and will be analyzed according to the actual treatment received.

Response rates with confidence intervals (CI) and *p*-values in both study arms will be estimated taking into account the two-stage nature of the design [23, 24]. Secondary endpoints will be evaluated descriptively. All toxicities will be summarized by relative and absolute frequency, and severity grade based on CTCAE V5.0. AE and SAE summary tables will provide the number and percentage of patients with AEs and the 95% Clopper-Pearson type CIs. All analyses will be done using SAS version 9.4 (SAS Institute, Cary, NC) or higher.

## Trial status

As of July 15th 2020, eight study sites are initiated. First initiation coincided with the beginning of the Covid-19 pandemic in Germany. Therefore, recruitment of patients was withheld. On May 8th 2020, recruitment was resumed after consultation with the ISMB. The first patient was enrolled on July 13th 2020.

## Discussion

In recent years, the concept of restoring the patients’ antitumor immunity by immune checkpoint inhibition has revolutionized cancer therapy, especially in advanced melanoma, renal carcinoma and NSCLC. Immune checkpoint molecules efficiently regulate T cell activation, and thus enable tumor cells to evade the immune system, for example by hijacking the PD-1/ PD-L1 interaction to downregulate effector T cells [25, 26]. To date, several human monoclonal antibodies pharmacologically blocking these interactions have been implemented in cancer therapy, such as the anti-PD-1 antibody pembrolizumab that has been approved in combination with chemotherapy for non-squamous NSCLC, irrespective of PD-L1 expression [27, 28].

Several studies have shown that immune checkpoint inhibition and radiotherapy in combination can act synergistically to further boost antineoplastic effects [29–32]. Although in a large phase III trial, no benefit of blockade of cytotoxic T lymphocyte-associated antigen 4 (CTLA-4) after radiotherapy was observed in metastatic prostate cancer [33], clinical trials, such as NICOLAS (NCT02434081) and DETERRED (NCT02525757), investigating concurrent PD-(L)1-directed immunotherapy and chemoradiotherapy in patients with locally advanced lung cancer, have confirmed efficiency at modest toxicity rates, particularly with respect to pneumonitis, which is one of the most threatening complications in NSCLC patients [34–36]. The PACIFIC trial showed an impressive improvement regarding PFS and OS with manageable side effects with durvalumab treatment after photon radiotherapy combined with chemotherapy in stage III, unresectable NSCLC patients, which has resulted in the approval of durvalumab consolidation after chemoradiotherapy [8, 9]. Safety of durvalumab in combination with concurrent palliative radiotherapy has also been confirmed in a smaller series of heterogeneous metastatic tumor patients [37]. Further, the combination may also prove to be an effective therapeutic concept in other entities, as for example is currently investigated in patients with head-and-neck cancer (NCT03283605) [38].

With regard to elderly and frail patients with unresectable stage III NSCLC unfit for chemotherapy, we hypothesize that durvalumab in combination with mildly hypofractionated TRT is safe and effective, given that feasibility and activity of this regime have been demonstrated in combination with chemotherapy in stage III patients [39, 40]. To date, no prospective trial has investigated such a therapeutic approach. Taking into account the concern of a cumulative risk for severe pneumonitis from applying both TRT and immunotherapy, the TRADE-hypo trial will investigate two radiation regimens combining durvalumab therapy with either conventionally fractionated or hypofractionated TRT.

The study design includes a safety stop-and-go phase preceding full enrollment to ensure that the hypofractionated treatment regimen can instantly be discontinued if deemed unsafe. Taking this and the interim efficacy analysis into account, this study is designed to efficiently avoid inadequate therapy and unnecessary costs. On the other hand, it may reveal a safe and efficient first-line immunoradiotherapeutic strategy for frail and elderly NSCLC stage III patients unable to receive chemotherapy, and, thus, provide an additional and optimized therapeutic option for this potentially undertreated patient cohort. Furthermore, data from this study may guide the design of larger, randomized trials investigating such an approach in this context.

In the accompanying translational research project, the host-tumor biology will be analyzed with a focus on immunological mechanisms responsible for antitumoral effects of both checkpoint inhibitors and radiotherapy, in an attempt to identify biomarkers predictive of therapy response. Eventually, this study may provide valuable data to explore how antitumoral immunological mechanisms may be activated in non-responders, currently representing about half the patients treated with immune-checkpoint inhibitors [27, 28, 41].

## Data Availability

Data generated by this study will be available for access from the corresponding author upon reasonable request.

## Abbreviations

AE: Adverse event
CCI: Charlson comorbidity index
CI: Confidence interval
CT: Computed tomography
CTCAE: Common Terminology Criteria for Adverse Event
CTLA-4: Cytotoxic T lymphocyte-associated antigen 4
CTV: Clinical target volume
ECG: Electrocardiogram
ECOG: Eastern Cooperative Oncology Group
eCRF: Electronic case report form
EOT: End of treatment
FU: Follow-up
GTV: Gross tumor volume
Gy: Gray
IP: Investigational Product
ISMB: Independent Safety Monitoring Board
ITT: Intent-to-treat
MRI: Magnetic resonance imaging
NSCLC: Non-small cell lung cancer
ORR: Objective response rate
OS: Overall survival
PD-1: Programmed cell death 1
PD-L1: Programmed cell death ligand 1
PFS: Progression-free survival
PP: Per-protocol
PTV: Planning target volume
QoL: Quality of life
RECIST 1.1: Response Evaluation Criteria in Solid Tumors, version 1.1
SAE: Serious adverse event
TRT: Thoracic radiation therapy
