# Correction: Thoracic radiotherapy plus Durvalumab in elderly and/or frail NSCLC stage III patients unfit for chemotherapy - employing optimized (hypofractionated) radiotherapy to foster durvalumab efficacy: study protocol of the TRADE-hypo trial

**DOI:** 10.1186/s12885-023-11270-x

**Published:** 2023-08-10

**Authors:** Farastuk Bozorgmehr, Inn Chung, Petros Christopoulos, Johannes Krisam, Marc A. Schneider, Lena Brückner, Daniel Wilhelm Mueller, Michael Thomas, Stefan Rieken

**Affiliations:** 1 Department of thoracic oncology, Thoraxklinik at University Hospital of Heidelberg, Röntgenstraße 1, Heidelberg, 69126 Germany; 2 Translational Lung Research Center Heidelberg TLRCH, Member of the german Center for Lung Research DZL, Im Neuenheimer Feld 156, Heidelberg, 69120 Germany; 3 Institute of Medical Biometry and Informatics, University Hospital of Heidelberg, Im Neuenheimer Feld 130.3, Heidelberg, 69120 Germany; 4 Translational Research Unit (STF), Thoraxklinik at University Hospital of Heidelberg, Röntgenstraße 1, Heidelberg, 69126 Germany; 5 Institute of Clinical Cancer Research IKF GmbH at Northwest Hospital, Steinbacher Hohl 2–26, Frankfurt am main, 60488 Germany; 6 Department of Radiation Oncology, University Medical Center Göttingen, Robert-Koch-Str. 40, Göttingen, 37075 Germany

**Correction: BMC Cancer 20, 806 (2020)**

10.1186/s12885-020-07264-8.

Following publication of the original article [1], the authors want to clarify the wording of two sentences.

In the line of the study procedures section “In total, 44 patients will be enrolled per group. After n = 18 patients have been enrolled to the HYPO- or CON-treatment arm, respectively, an interim efficacy analysis for the respective arm will be conducted based on the objective response rate (ORR) at 12 weeks after first durvalumab administration”, the sentence was refined to “[…] the objective response rate (ORR), **when the 18th patient in each arm has undergone first radiographic assessment (**at 12 weeks after first durvalumab administration**)**.”

In the study endpoint section, in the line “The ORR evaluated 12 weeks after first durvalumab administration (according to RECIST 1.1) is set as the primary efficacy endpoint.”, the sentence changed to “The ORR according to RECIST 1.1 is set as the primary efficacy endpoint.”
